# microRNA-34a-Mediated Down-Regulation of the Microglial-Enriched Triggering Receptor and Phagocytosis-Sensor TREM2 in Age-Related Macular Degeneration

**DOI:** 10.1371/journal.pone.0150211

**Published:** 2016-03-07

**Authors:** Surjyadipta Bhattacharjee, Yuhai Zhao, Prerna Dua, Evgeny I. Rogaev, Walter J. Lukiw

**Affiliations:** 1 LSU Neuroscience Center, Louisiana State University Health Science Center, New Orleans, LA, 70112, United States of America; 2 Department of Anatomy and Cell Biology, Louisiana State University Health Science Center, New Orleans, LA, 70112, United States of America; 3 Louisiana State Technical University, Ruston, LA, 71270, United States of America; 4 Department of Psychiatry, Brudnick Neuropsychiatric Research Institute, University of Massachusetts Medical School, Worcester, MA, 01604, United States of America; 5 Department of Genomics and Human Genetics, Laboratory of Evolutionary Genomics, Vavilov Institute of General Genetics, Russian Academy of Sciences, Moscow, 119991, Russia; 6 Faculty of Bioengineering and Bioinformatics, Lomonosov Moscow State University, Moscow, 119234, Russia; 7 Department of Ophthalmology, Louisiana State University Health Science Center, New Orleans, LA, 70112, United States of America; 8 Department of Neurology, Louisiana State University Health Science Center, New Orleans, LA, 70112, United States of America; Indiana University College of Medicine, UNITED STATES

## Abstract

The aggregation of Aβ42-peptides and the formation of drusen in age-related macular degeneration (AMD) are due in part to the inability of homeostatic phagocytic mechanisms to clear self-aggregating Aβ42-peptides from the extracellular space. The triggering receptor expressed in myeloid/microglial cells-2 (TREM2), a trans-membrane-spanning, sensor-receptor of the immune-globulin/lectin-like gene superfamily is a critical component of Aβ42-peptide clearance. Here we report a significant deficit in TREM2 in AMD retina and in cytokine- or oxidatively-stressed microglial (MG) cells. RT-PCR, miRNA-array, LED-Northern and Western blot studies indicated up-regulation of a microglial-enriched NF-кB-sensitive miRNA-34a coupled to a down-regulation of TREM2 in the same samples. Bioinformatics/transfection-luciferase reporter assays indicated that miRNA-34a targets the 299 nucleotide TREM2-mRNA-3’UTR, resulting in TREM2 down-regulation. C8B4-microglial cells challenged with Aβ42 were able to phagocytose these peptides, while miRNA-34a down-regulated both TREM2 and the ability of microglial-cells to phagocytose. Treatment of TNFα-stressed MG cells with phenyl-butyl nitrone (PBN), caffeic-acid phenethyl ester (CAPE), the NF-B-inhibitor/resveratrol analog CAY10512 or curcumin abrogated these responses. Incubation of anti-miRNA-34a (AM-34a) normalized miRNA-34a abundance and restored TREM2 back to homeostatic levels. These data support five novel observations: **(i)** that a ROS- and NF-B-sensitive, miRNA-34a-mediated modulation of TREM2 may in part regulate the phagocytic response; **(ii)** that gene products encoded on two different chromosomes (miRNA-34a at chr1q36.22 and TREM2 at chr6p21.1) orchestrate a phagocytic-Aβ42-peptide clearance-system; **(iii)** that this NF-kB-mediated-miRNA-34a-TREM2 mechanism is inducible from outside of the cell; **(iv)** that when operating normally, this pathway can clear Aβ42 peptide monomers from the extracellular medium; and **(v)** that anti-NF-kB and/or anti-miRNA (AM)-based therapeutic strategies may be useful against deficits in TREM-2 receptor-based-sensing and -phagocytic signaling that promote pathogenic amyloidogenesis.

## Introduction

Currently affecting about 150 million individuals worldwide, age-related macular degeneration (AMD) is a common, neurodegenerative disorder of the human retina characterized clinically by the progressive erosion of central vision [1,2]. AMD is further subdivided into a “wet” form, involving choroidal neovascularization, and the much more common "dry" form of AMD, characterized by the presence of yellow lipoprotein-rich deposits, called drusen, in the macula, the central portion of the retina. The drusen of AMD typically develop with aging and contain a beta-amyloid precursor protein (βAPP)-derived 42 amino acid amyloid beta peptide (Aβ42) as a major component [3–5]. The molecular-genetic mechanisms regulating Aβ42 peptide accumulation and clearance are not completely understood, but appear to involve a receptor-mediated sensing of Aβ42 peptide monomers and other toxic molecules in the extracellular space as an initial step in phagocytosis and homeostatic clearance. One prominent sensor-receptor for Aβ42-peptide clearance in the CNS is the triggering receptor expressed in myeloid/microglial cells-2 (TREM2; chr6p21.1), a ~230 amino acid, single pass type 1 transmembrane sensor-receptor protein enriched in the plasma membrane of microglial (MG) cells [6–11]. Mutations and loss-of-function for TREM2 have been associated with deficiencies in phagocytosis, the innate-immune system, axonal and synaptic abnormalities, amyloidogenesis and progressive dementia in progressive neurological diseases of the human CNS including *polycystic lipomembranous osteodysplasia with sclerosing leukoencephalopathy (PLOSL;* also known as Nasu-Hakola disease [6–11] as well as more recently in sporadic amyotrophic lateral sclerosis (ALS) [11] and in Alzheimer’s disease (AD) [6–15].

Micro RNAs (miRNAs) are ~22 nucleotide, non-coding RNA single stranded (ssRNA) molecules that represent a family of heterogeneous, evolutionarily conserved, regulatory RNAs that recognize the 3’ un-translated region (3’UTR) of specific messenger RNA (mRNA) targets [16,17]. In doing so miRNAs down-regulate the post-transcriptional stability or translational efficiency of their target mRNAs, thus functioning as natural negative regulators of gene expression [16–19]. Of the ~2650 human miRNAs so far identified: **(i)** only a specific subset of miRNAs are highly expressed in the CNS; **(ii)** many of these are critical to the regulation of normal brain and retinal cell function in health and aging; and **(iii)** many of these miRNAs appear to be inducible by age-related pathological and environmental factors [17–21]. Like neurons and astroglia, MG cells express a select family of miRNAs that support homeostatic retinal gene expression functions and specific miRNA abundances and are significantly altered in AMD-affected retina when compared to age-matched controls [20–24]. As few miRNAs have been functionally linked to specific retinal pathways involving phagocytosis, these studies were undertaken to further understand the involvement of specific, retinal-enriched, inducible miRNAs in the molecular-genetic mechanism that drives amyloidogenesis, TREM2 down-regulation, drusen formation and AMD-type change. Here we provide evidence that in human AMD and stressed MG cells there occurs a selective up-regulation of an inducible, NF-kB-regulated miRNA-34a coupled to a significant down-regulation of TREM2 expression. Bioinformatics and transfection experiments indicate that miRNA-34a targets the 299 nucleoide (nt) TREM2 mRNA 3’UTR and significantly down-regulates TREM2 expression in MG cells. Transfection of C8B4 MG cells using TREM2 3’UTR luciferase reporter constructs showed specific and significant interaction with miRNA-34a but not with other scrambled (**SC**) and/or negative control (**NC**) miRNA-34a sequences. Further, up-regulation of the pro-inflammatory miRNA-34a and TREM2 down-regulation was also observed in reactive oxygen species (ROS)-, IL-1β- and TNFα-stressed MG cell cultures, an effect that could be significantly reversed using anti-ROS, anti-NF-kB and/or anti-miRNA-34a therapeutic strategies. These studies provide 5 new observations: **(i)** that gene products encoded on two different chromosomes (miRNA-34a at chr1q36.22 and TREM2 at chr6p21.1) coordinate a phagocytosis-mediated Aβ42-peptide clearance system in stressed-MG cells and in sporadic AMD retina; **(ii)** that increased expression of a ROS-induced, inflammatory cytokine and NF-kB-sensitive MG-enriched miRNA-34a down-regulates a specific mRNA target encoding TREM2, a membrane-spanning glycoprotein known to contribute to the clearance of pro-inflammatory Aβ42 peptides; **(iii)** that a miRNA-34a-regulated TREM2 circuit can clear low molecular weight Aβ42 peptides from the extracellular medium; **(iv)** that this miRNA-34a-TREM2 clearance system is inducible from outside of the cell; and **(v)** that anti-ROS, anti-miRNA or anti-NF-kB pharmacological strategies may be useful in the clinical management of deficits in phagocytic signaling that drive pathogenic amyloidogenesis. These findings suggest that an epigenetic mechanism involving an NF-кB-mediated, miRNA-34a-regulated down-regulation of TREM2 expression may shape innate-immune and phagocytic responses that contribute to amyloidogenesis and inflammatory neurodegeneration characteristic of the AMD process.

## Materials and Methods

### Human Retinal Tissues

**Fig 1:**
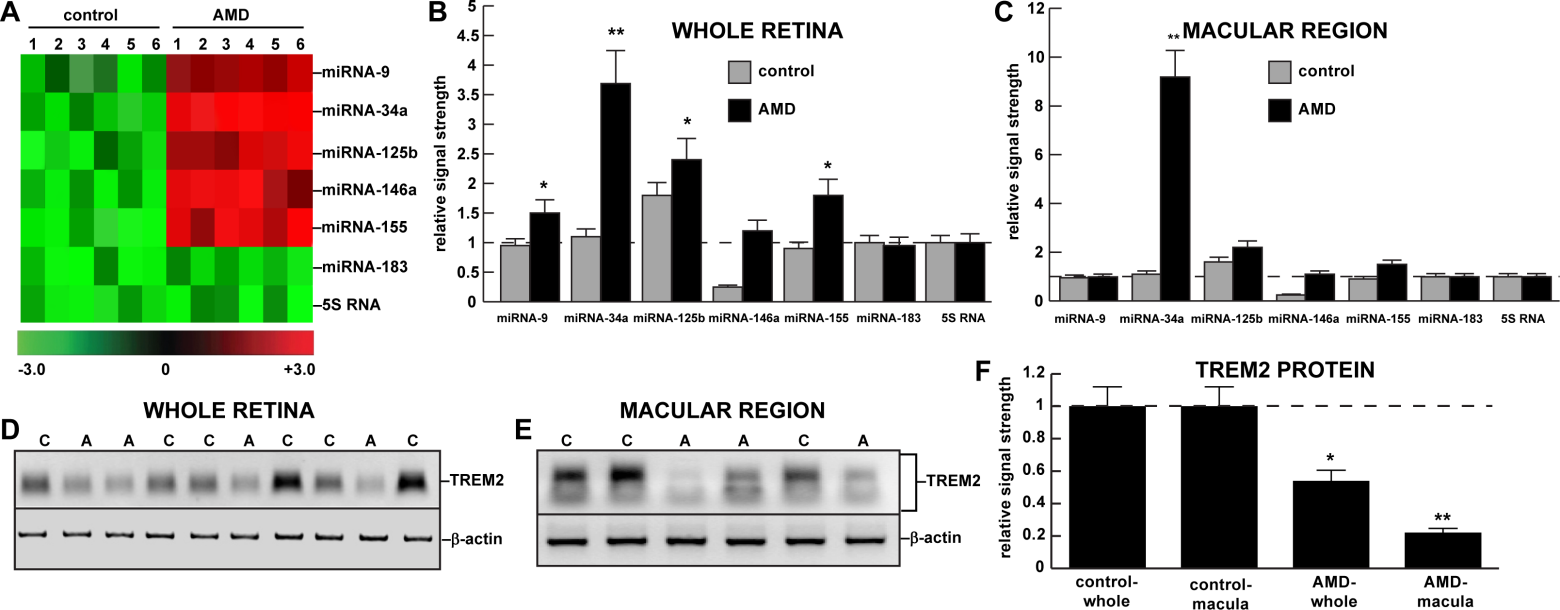
**Up-regulation of miRNA-34a and down-regulation of TREM2 in AMD whole retina and macular region versus age-matched controls; (A)** color-coded cluster diagram; miRNA-34a and miRNA-155 showed the greatest up-regulation to 3.3- and 1.8-fold over their respective controls in whole retina and 8.8- and 1.6-fold over their respective controls in the macular region; a significant up-regulation was not observed for either miRNA-183 or 5S RNA controls in either **(B)** whole retina or **(C)** the macular region; one preliminary study involving miRNA-34a up-regulation and a miRNA-34a-mediated TREM2 down-regulation has previously been reported for the hippocampal CA1 region of AD brain [10,12]; miRNA-34a is part of an inducible pro-inflammatory miRNA quintet consisting of miRNA-9, miRNA-34a, miRNA-125b, miRNA-146a, miRNA-155 involved in degeneration in the human CNS; there were no significant differences between age for control or AMD tissues; all AMD cases were for moderate-to-advanced stages of disease; all post-mortem intervals were 2.5 h or less [20–22]; there were no significant differences in RNA quality (all RNA integrity numbers—RIN values—were 8.1–9.1) or yield between the control (N = 9) or AMD (N = 12) groups (*p* >0.05, ANOVA); **(D-F**) Western blot of TREM2 protein in the same AMD (**A**) and control (**C**) tissues in **(D)** whole retina and **(E)** the macular region; in whole retina (N = 10) TREM2 protein levels were reduced to 0.54-fold of control levels and in the macular region (N = 5) TREM2 levels were reduced to 0.22-fold of control; **(E)** note that TREM2 Western blot analysis on **≤**10% TGSDS gels show multiple bands due to the variable post-translational glycosylation of ~25kDa core TREM2 protein (unpublished); TREM2 protein levels are shown as the mean plus one standard deviation (SD) are bar-graphed in **(F)**; see text; *p<0.05; **p<0.001 (ANOVA).

Human post-mortem retinal tissues and/or total RNA and/or total protein extracts from human retina were obtained from archived material at the LSU Neuroscience Center, New Orleans LA, from the Southern Eye Bank, Metairie LA or from commercial sources; all AMD samples obtained from dry AMD tissues were used in accordance with the institutional review board (IRB)/ethical guidelines at the LSU Health Sciences Center where these studies were approved [22]. The mean age +/- one standard deviation of the control and AMD retinal tissues were 70.1+/-8.5 and 72.3/-7.5 years respectively, the age range was 65–78 years for all tissues, and all retinal samples were from female Caucasians. Because post-mortem interval (PMI) is a factor that can affect RNA stability and quality, all RNAs were derived from tissues having a PMI of ≤2.5 hrs; the RNA A_**260/280**_ ranged between 2.04 and 2.14 for all samples; the RNA integrity number for each sample was 8.5 or greater (**Fig 1**) [19,20,22,23,25–27].

### C8B4 microglial (MG) Cells in Culture

C8B4 murine microglial (MG) cell lines expressing the classical MG markers MAC1, F4/80, 2-4G2, Iba1 and TREM2 (but not GFAP) were cultured according to the manufacturer’s protocols (ATCC CRL-2540; Manassas VA) and previously published peer-reviewed work from our lab [12,19,21,28,29]. MG cell cultures were used at 3 days or 1 wk, and contained approximately 95% MG cells and 5% astroglial/oligodendroglial cells. They were prepared and analyzed according to established methods by the ATCC or our own laboratory [19,26,27,29].

### MG Cell Transfection

**Fig 2:**
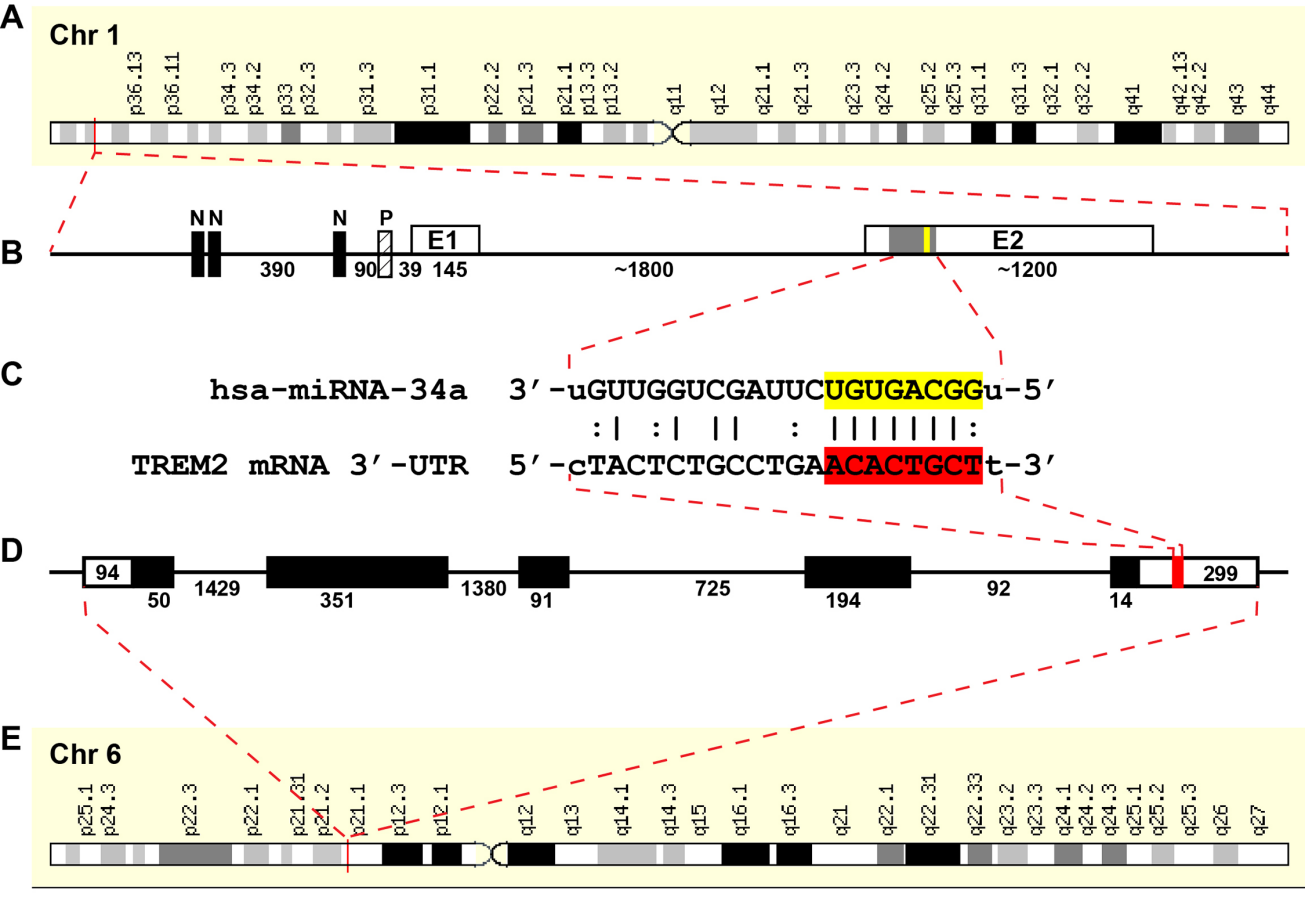
**(A) Schematic complementarity map** for a micro RNA-34a-TREM2 mRNA-3’-untranslated region (hsa-miRNA-34a-TREM2-mRNA-3’UTR) interaction between primary gene products on chromosome 1 and 6 [12]; human sequences shown; not drawn to scale; **(B)** hsa-miRNA-34a, encoded chr1p36.15 contains 3 canonical NF-kB sites **(N)** in the upstream promoter (http://www.genecards.org/cgi-bin/carddisp.pl?gene=MIR34A) [8,12,56];; E1 = exon 1; E2 = exon 2 of the miRNA-34a gene; miRNA-34a expression is known to be NF-kB-sensitive in human brain cells [12,58]; **(C)** miRNA-34a precursor is processed into a mature 22 nucleotide hsa-miRNA-34a sequence; the free energy of association (E_A_) between hsa-miRNA-34a and the TREM2 mRNA-3’UTR sequence is ~16.2 kcal/mol; the miRNA-34a seed sequence **3’-UGUGACGG-5’** is overlaid in yellow; the complementary TREM2-3’-UTR recognition (DNA) sequence **5’-ACACTGCT-3’** is overlaid in red; an ‘**|**’ indicates a full hydrogen bond between miRNA-34a and the TREM2-mRNA-3’UTR and a ‘:’ indicates a partial hydrogen bond; **(D)** the hsa-miRNA-34a recognition feature within the TREM2-3’UTR is located about midway in the 299 nucleotide (nt) TREM2-3’UTR; several other brain-enriched miRNAs located within the TREM2-3’UTR and may also affect TREM2 mRNA stability and regulate its expression; sequence structures in **(B)** and **(D)** are not drawn to scale; **(E)** TREM2 is encoded as a single copy gene at human chr6p21.1; the primary transcript is a 2.7k nt TREM2 mRNA (http://www.genecards.org/cgi-bin/carddisp.pl?gene=TREM2) with a half-life of about 20 hr [60]; it is noteworthy that the TREM2 gene has no strong NF-kB binding site within at least 11 kb of its transcription start site and NF-kB activation has no strong effects on TREM2 transcription (unpublished); single stranded ribonucleotide sequences and alignment derived using miRBASE algorithms (European Bioinformatics Institute, Wellcome Trust Genome Campus, Hinxton UK; srv/microcosm/cgi-bin/targets/v5/ detail_view.pl? transcript_id = ENST00000 373113) [3–6,8,12,20,56,58].

**Fig 3:**
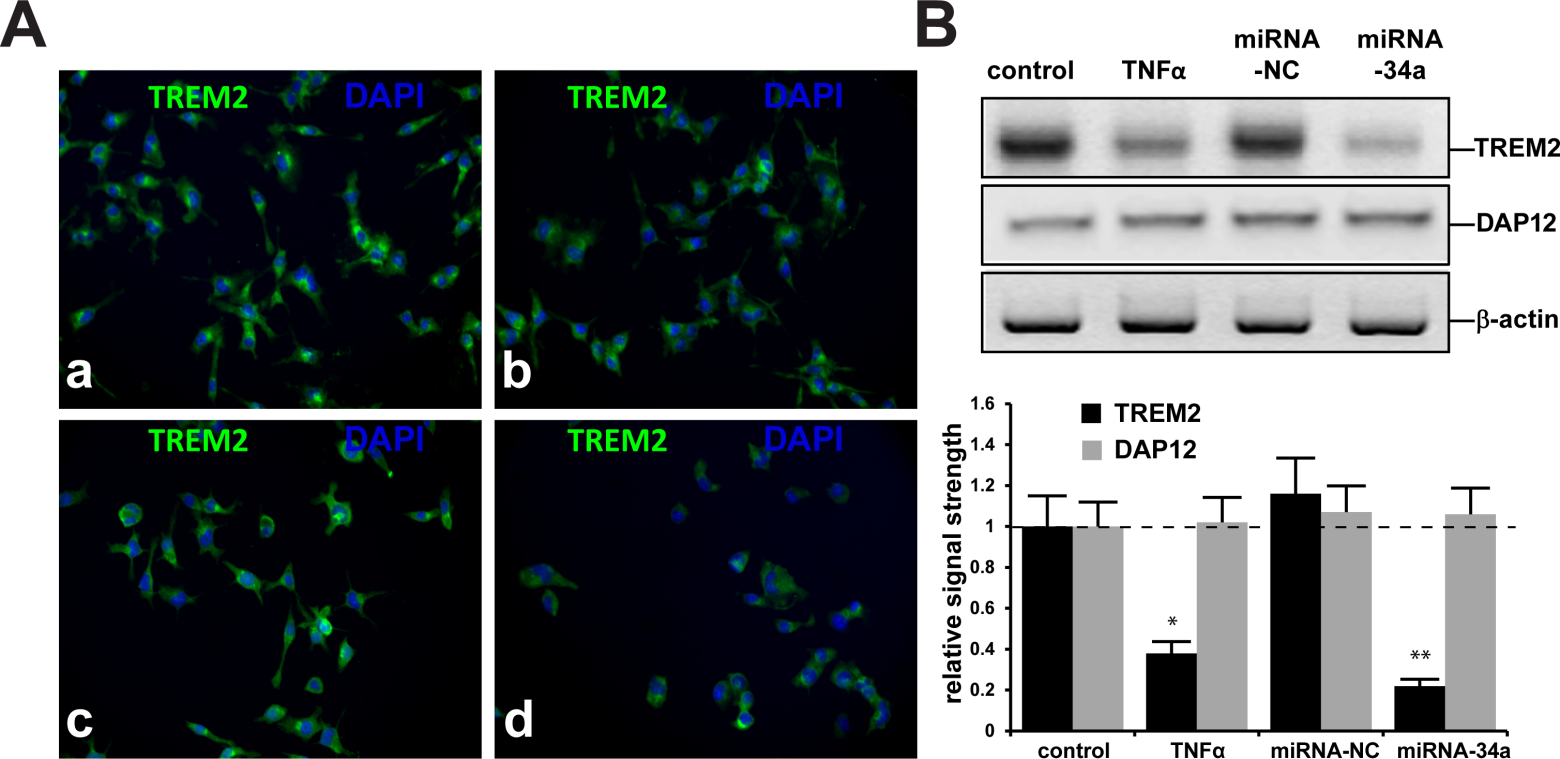
**TREM2 and DAPI nuclear staining of C8B4 murine microglial (MG) cells (A): (a)** control MG cells cultured 3 days, magnification 20x; **(b)** treated with TNFα; **(c)** treated with 50 nM miRNA-34a-sc (24 hr) **or (d)** treated with 50 nM miRNA-34a (24 hr); note significantly reduced TREM2 protein signals in stressed MG cells **(b** and **d)** compared to control (**a**) or miRNA-34a-sc-treated MG cells (**C**); Westerns blots were performed for TREM2 using an antibody directed against the 277 amino acid murine TREM2 (M227;sc-48765) or TYROBP (DAP12; C-20; sc-7853); SCBT, Santa Cruz, California, USA); nuclei stained with DAPI as in **(Fig 6); (B) (upper panel)** representative Western blot and **(B) (lower panel)** bar graph analysis of TREM2 and DAP12 protein levels in control, TNFα-, miRNA-NC or miRNA-34a-stressed MG cells; in this sample set TREM2 protein levels were found to be significantly reduced in TNFα- or miRNA-34a-treated MG cells compared to age matched controls; there were no significant differences in the abundance of the TYROBP/DAP12 adaptor protein amongst control, TNFα, miRNA-NC or miRNA-34a treated cells (**Fig 7**); N = 8; *p<0.05, **p<0.01 (ANOVA).

**Fig 4:**
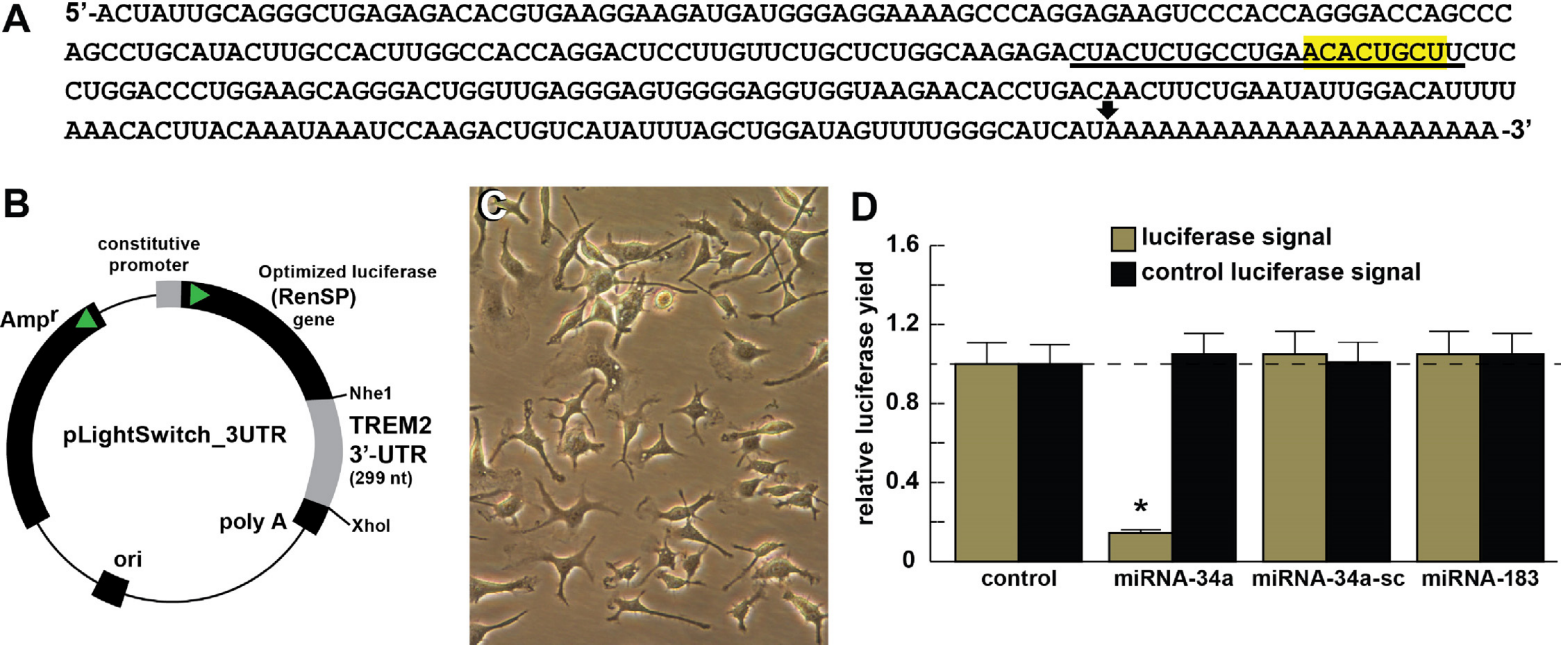
Functional validation of a miRNA-34a-TREM2–3’UTR interaction. **(A)** ribonucleotide sequence of the 299 nt TREM2-mRNA-3’-UTR is shown in the 5’-3’ direction; the 22 nt miRNA-34a-TREM2 3’UTR complementarity-interaction region is indicated by a black underline and the 8 nt TREM2-mRNA-3’-UTR seed sequence **5’-ACACUGCU-3’** is overlaid in yellow; a single arrowhead indicates the 5’ end of a poly A+ tail in the TREM2 mRNA (22 ‘A’ nt shown; the length of this poly A+ tail is variable); TREM2 mRNA sequence derived from NM_018965; TREM2 transcript is the major X1 variant (see also http://switchdb.switchgeargenomics.com/productinfo/id_801321/) (**Fig 3**); **(B)** TREM2-mRNA-3’UTR expression vector luciferase reporter assay (pLightSwitch-3’UTR; Cat#S801178; Switchgear Genomics, Palo Alto CA); in this vector, the entire 299 nucleotide TREM2 3’UTR was ligated into the unique Nhe1-Xho1 site; **(C)** control C8B4 murine microglial cells, 1 week in culture; phase contrast bright field microscopy 20x; C8B4 cells transfected with the TREM2-mRNA-3’UTR expression vector luciferase reporter were treated exogenously with miRNA-34a, a scrambled control miRNA-34a (miRNA-sc) or control miRNA-183; see references and text for further details [18,19]; **(D)** compared to control, C8B4 cells transfected with a scrambled (**sc**) control pLightSwitch-3’UTR vector, the TREM2-mRNA-3‘UTR vector exhibited decreased luciferase signal to a mean of 0.16-fold of controls in the presence of miRNA-34a; this same vector exhibited no change in the presence of the control miRNA-34a-sc or miRNA-183; for each experiment (using different batches of MG cells) a control luciferase signal was generated and included separate appropriate controls with each analysis; in addition a control vector β-actin-3’UTR showed no significant effects on the relative luciferase signal yield after treatment with either miRNA-183 or miRNA-34a (data not shown); dashed horizontal line set to 1.0 for ease of comparison; N = 5; *p<0.001 (ANOVA). The results suggest a physiologically relevant miRNA-34a- TREM2-mRNA-3‘UTR interaction and a miRNA-34a-mediated down-regulation of TREM2 expression in stressed MG cells. This pathogenic ineraction may be related to the down-regulation of other immune system genes by up-regulated pro-inflammatory miRNAs in the CNS [12,19,22] and/or an impairment in cellular phagocytosis or related phagocytic signaling [5–7,20,21].

**Three** day and 1 week old MG cells were transfected with a TREM2-mRNA-3’UTR expression vector luciferase reporter assay (pLightSwitch-3’UTR; cat#S801178; see **Figs 2, 3 and 4**) containing the entire 299 bp TREM2-mRNA-3’UTR, with miRNA-34a mimics or with other miRNA-related sequences following the manufacturer’s instructions and as previously described (Switchgear Genomics, Palo Alto CA) [12,18–21,29]. After various treatment conditions as indicated, cells were processed for luciferase assay using an established luciferase reporter assay kit as previously described in detail in the manufacturer’s protocol or in earlier reports from our laboratory (Dual Luciferase System, Promega) [12,18–21,29].

### miRNA isolation from human tissues and MG cells

In human retinal tissue studies 10 mg wet weight samples were isolated from the whole retina and/or the macular region from dry AMD patients or from age-matched controls. In MG cell studies cells from 3 to 5 ~50–60% confluent 3.5 cm diameter 6-well CoStar plates were scraped, taken up into a 20 ml syringe and RNAse and DNAse-free, DEPC-treated plasticware and gently packed using centrifugation [20,25,29]. For both tissues and cells a guanidine isothiocyanate- and silica gel-based membrane total RNA purification system was used to isolate total RNA (chiefly miRNA, tRNA, 5SRNA, mRNA and rRNA) from each sample [12,22,29]. A miRNA isolation kit (PureLink™ Invitrogen, Carlsbad, CA) was further used to isolate and enrich miRNA from total RNA samples. Total RNA concentrations were quantified using RNA 6000 Nano LabChips and a 2100 Spectral Bioanalyzer (Caliper Technologies, Mountainview, CA; Agilent Technologies, Palo Alto, CA) and typically yielded about 1.1–1.6 μg of very high quality total RNA per mg wet weight of tissue.

### microRNA arrays, RT-PCR and brain-enriched miRNAs

As a preliminary screen, and to obtain general trends for miRNA abundance and speciation, total miRNA was pooled and analyzed as an AMD group (N = 12) and an age-matched control group (N = 9) using commercially available biofluidic miRNA arrays (~2650 human miRNAs analyzed; LC Sciences, Houston TX); (**Fig 1**). Specific miRNAs showing strong and significant hybridization signals in AMD or controls were studied further. Subsequently, DNA targets for human miRNA-9, miRNA-34a and 5S ribosomal RNA (5SRNA) and miRNA-183 controls were spotted onto GeneScreen Plus nylon membranes using a Biomek® 2000 laboratory automation workstation (Beckmann, Fullerton, CA); these mini-miRNA array panels were cross-linked, baked, hybridized and probed according to the manufacturer’s protocol (NEN® Research Products, Boston MA) [20–22]. Every second mini-miRNA array panel generated was normalized by probing with purified single radiolabelled ssRNA or miRNA (miRNA-9, miRNA-34a, miRNA-125b, miRNA-146a, miRNA-155 miRNA-183 and/or 5S RNA) to ascertain equivalent 5SRNA and individual miRNA loadings. Mini-miRNA panels were next probed with total labeled miRNAs isolated from AMD or age-matched controls. AMD or control extracts (25 μg) containing miRNA or 5SRNA (5μg) were spotted onto GeneScreen membranes, cross-linked, baked, hybridized and probed with specific DNA oligomers corresponding to specific miRNAs), radiolabeled using [-^**32**^P]-δATP (6000 Ci/mmol) and a T4 polynucleotide kinase labeling system (Invitrogen) [22,29]. RT-PCR was performed using techniques previously described [17–23,29].

### ROS, IL-1β, TNFα, PBN, curcumin, PDTC, CAY10512 or CAPE treatment of MG cells

**Fig 5:**
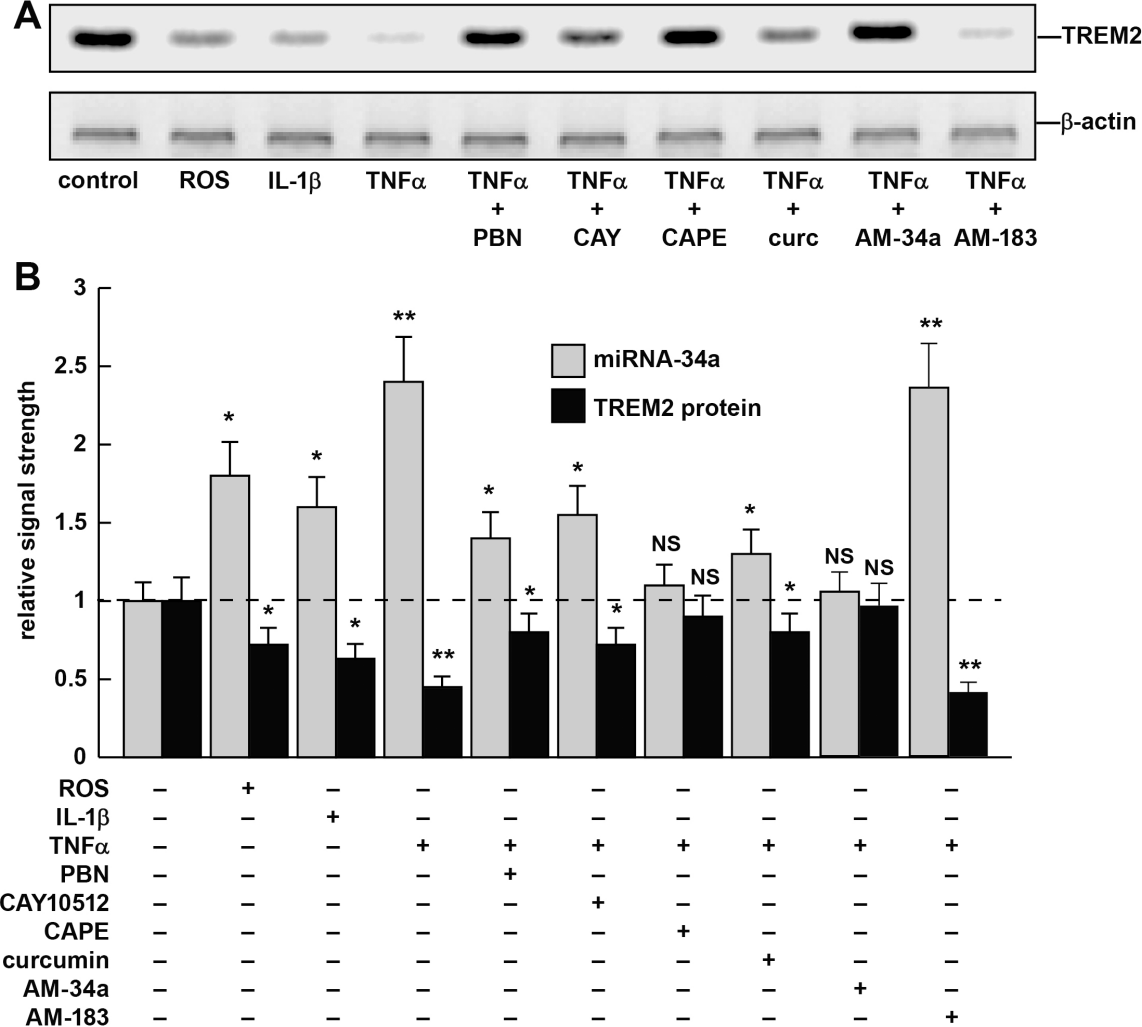
**Reactive oxygen species (ROS)-, IL-1β- or TNFα-stressed MG cells—involvement of NF-kB and miRNA-34a and the effects of NF-kB inhibitors or anti-miRNAs (AMs)–(A)** representative Western blot of TREM2 protein levels in variably stressed MG cells both in the presence and absence of NF-kB and AMs; miRNA-34a levels were determined using microfluidic miRNA array analysis in the same sample (**Fig 1**); **(B)** note ROS-, IL-1β- or TNFα-induced increases in miRNA-34a and TREM2 protein decreases in the same sample; when present the antioxidants and/or NF-kB inhibitors PBN, CAY10512, CAPE or curcumin quenched this induction; see text for further details; similarly anti-miRNA-34a (AM-34a) but not 4 other AM species: AM-183 (or AM-9, AM-125b or AM-146a; data not shown) selectively lowered miRNA-34a levels while increasing TREM2 to 0.92 of control levels; N = 6; *p<0.05, **p<0.01 (ANOVA); NS = not significant.

To induce ROS a hydrogen peroxide solution (H_2_O_2_; 30 wt % in H_2_O; Sigma-Aldrich, St. Louis MO) was used at 2.0 uM concentration for 1 hr in the MG cell cultures. Similarly IL-1β (I4019; Sigma-Aldrich; 10 ng/ml cell medium), TNFα (T7539; Sigma-Aldrich; 5 ng/ml), N-tert-butyl-α-phenyl nitrone (phenyl butyl nitrone; PBN; B7263; Sigma Aldrich; 10 mM) or curcumin (diferuloyl-methane; C7727; Sigma Aldrich; 10 uM) was used as previously described (**Figs 3, 4 and 5**) [22,29–35]. Locked nucleic acids (LNA) and anti-miRNA (AM-34a, AM-183) were purchased from Ambion (Invitrogen, Carlsbad CA). A 22 oligonucleotide anti-miRNA-34a (AM-34a; 5’-TCTTCCTGCT TTGTCTCTGCCT-3’) or anti-miRNA-183 (AM-183; 5’-AGTGAATTCTACCAGTGCCATA-3’) were used at 5–20 nM concentrations, in one week old MG cells for a total treatment time of 36 hrs after H_2_O_2_, IL-1, and/or TNFα induction. As required, one week old MG cells were treated with the free radical scavenger and antioxidant phenyl-butyl nitrone (PBN), the polyphenolic trans-stilbene resveratrol analog CAY10512, the synthetic, anti-inflammatory bee resin-derived NF-kB inhibitor caffeic acid phenethyl ester (CAPE) or the natural, anti-inflammatory diarylheptanoid curcumin just prior to the addition of the NF-B-containing pre-miRNA-34a promoter and luciferase reporter vector (**Figs 4 and 5**).

### Murine MG cell mediated phagocytosis of Aβ42 peptides

**Fig 6:**
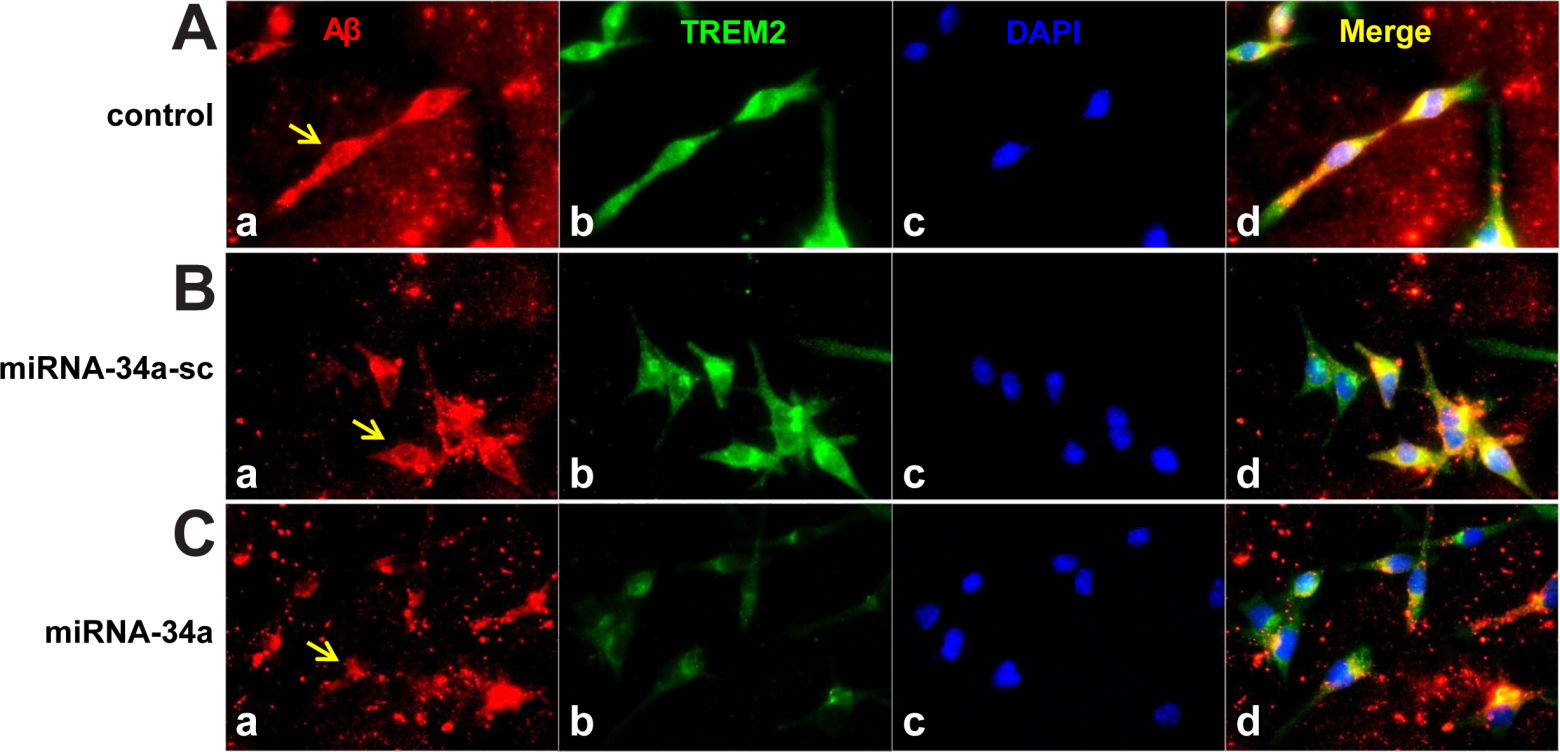
**Control C8B4 murine MG cells efficiently phagocytose Aβ42 peptides; miRNA-34a treated MG cells do not; (A)** C8B4 MG cells (ATCC CRL-2540) were cultured for 3 days (control **a-d**, top row); cells were treated with 5 μM of Aβ42 for 24 hr before staining; Aβ42 peptide (American Peptide Company, Sunnyvale, CA, cat # 62-0-80A); Aβ42 peptide was prepared as previously described [33]; briefly, Aβ42 peptides were initially solubilized in hexafluoroisopropanol (HFIP; Fluka Chemical, cat# 52512; Sigma-Aldrich, St. Louis MO), aliquoted, and stored at −20°C as an HFIP film. After vacuum evaporation of HFIP, aliquoted peptide was re-suspended with DMSO to 5 mM and diluted to 5 μM into the cell culture media; **(B)** cells were treated with a scrambled miRNA-34a sequence (miRNA-34a-**sc**; 30 nM;, control, **a-d**, middle row); or **(C)** with an LNA-stabilized miRNA-34a (30 nM; miRNA-34a stressed; **a-d**; bottom row); treatments were for 24 hr before incubation with 5 uM of Aβ42 (made up as in [33]) for another 24 hr before assay; MG cells were subsequently stained using a murine amyloid beta MABN10 (red fluorescence λ_max_~650 nm; anti-Aβ antibody, clone W0-2; Millipore, Bellerica MA), a TREM-2 antibody (M-227): sc-48765 (green fluorescence; λ_max_~510 nm; Santa Cruz, Santa Cruz CA) or DAPI nuclear stain (blue fluorescence; λ_max_~470 nm) as in **Fig 3;** arrows indicate Aβ42 uptake into MG cells; note decreased presence of TREM2 in miRNA-34a treated MG cells (bottom row, panel B) and decrease in ingested Aβ42 peptide within C8B4 cells (**C**; bottom row, panel **d**). Taken together these results support a miRNA-34a-mediated impairment of sufficient TREM2 to phagocytose Aβ42 peptide from the extracellular space; note self-aggregation of Aβ42 peptide after 24 hrs and Aβ42 peptide affinity for TREM2 containing cells (leftmost panels) and internalization (rightmost panel; yellow merge; λ_max_~580 nm); magnification 20x; Aβ peptide quantification was performed using SlideBook 5.0 (Intelligent Imaging Innovations) and ImageJ (NIH) software; under these conditions about 42% of externalized Aβ42 was cleared; additional relevant methods have been described [10,44].

C8B4 MG cells were treated with 5 μM of Aβ42 for 24 hr before staining (American Peptide Company, Sunnyvale, CA, cat # 62-0-80A) (**Figs 5 and 6)**. Samples of this Aβ42 peptide when analyzed on Western blots were comprised mostly of monomer, however after 24 hrs dimers and larger Aβ42 aggregates were present [31–33]. MG cells were subsequently stained using a murine amyloid beta MABN10 (red fluorescence λ_max_~650 nm; anti-Aβ antibody, clone W0-2; Millipore, Bellerica MA), a TREM-2 antibody (M-227): sc-48765 (green fluorescence; λ_max_~510 nm; Santa Cruz, Santa Cruz, CA) and/or DAPI nuclear stain (**Fig 6**). MG cells were imaged using a Zeiss Axioplan 2 deconvolution microscope (Carl Zeiss Microscopy, Göttingen, Germany).

### Western blot analysis of Aβ42 peptides, TREM2, DAP12 and β-actin in brain tissues and MG cells

Western blots were performed for identification and quantiation of Aβ42 peptide species, TREM2 and β-actin protein using human- and/or murine-specific primary antibodies directed against a murine anti-Aβ antibody MABN10 (red fluorescence λ_max_~650 nm; anti-Aβ antibody, clone W0-2; Millipore, Bellerica MA), the control protein marker β-actin (3598–100; Sigma-Aldrich Chemical Company, St Louis, Missouri, USA), human or murine TREM2 (B3; sc-373828, H160; sc-49764 or M227; sc-48765; and DAP12 Antibody (C-20): sc-7853; Santa Cruz Biotechnologies, Santa Cruz CA; [12,22,29,33]. When used ELISA analysis of mouse TREM2 utilized a mouse TREM2 sandwich ELISA kit according to the manufacturer’s protocols (abx154806; detection limit ~75 pg/ml; Abbexa Cambridge UK/NeoScientific Woburn MA, USA; or LS-F7884 ELISA Kit, LifeSpan Biosciences Seattle WA, USA).

### Statistical analysis of data and interpretation

An unchanging miRNA-183 and the abundant 107 nucleotide 5S ribosomal RNA (5SRNA) marker were used as non-coding ssRNA internal controls for miRNA determinations. Relative miRNA and TREM2 mRNA signal strengths were quantified against miRNA-183 and/or 5SRNA in each sample using data-acquisition software provided with a GS250 molecular imager (Bio-Rad, Hercules, CA). Graphic presentations were performed using Excel algorithms (Microsoft, Seattle, WA) and Adobe Photoshop 6.0 (Adobe Systems, San Jose CA). In this paper statistical significance was analyzed using either a Student's t-test or a two-way factorial analysis of variance (*p*, ANOVA; SAS Institute, Cary, NC). A *p<0*.*05* was deemed as statistically significant; all experimental values were expressed as means +/- one standard deviation (SD) of that mean (**Figs 1 and 3–5**).

## Results

### Human retinal tissues—case selection and total RNA quality

PMIs for age-matched control or AMD human retinal tissues were all ≤2.5 hr; age-matched control or AMD sample tissues exhibited no significant differences in age, PMI, RNA A_**260/280**_ indices or RNA integrity numbers (RIN), age-matched control versus AMD. We also noted no significant differences in total RNA purity or yields between the control and AMD groups for any tissues analyzed in this study.

### Relative abundance of miRNA-34a and TREM2 in control and AMD retina

miRNA arrays were initially used to screen for relative miRNA abundance in adult control whole retina, and then quantitative differences between adult control and AMD miRNA in the the macular region were further examined [20,26] (**Fig 1A–1C**). Similar to previous reports of total miRNA abundance showed relative abundance in control retina was 5SRNA>>miRNA-125b>>miRNA-34a>>miRNA-146a>>miRNA-155>>miRNA-183 both in adult control and AMD retinal tissue samples. However, miRNA-34a in the retina exhibited up to a 6.3-fold increases AMD over control in advanced AMD. Although relatively abundant in the human retina, 5SRNA, and the much less abundant miRNA-183 exhibited no significant change in relative abundance among any of the samples tested. Of the 5 miRNAs found to be significantly up-regulated on these panels (**Fig 1**) miRNA-34a and miRNA-146a showed the greatest up-regulation in AMD ranging between 2.1- to 6.3-fold over age-matched controls (*p*<0.01, ANOVA). Interestingly all these 5 miRNAs have been extensively studied and previously categorized as being inducible and regulated by the pro-inflammatory transcription factor NF-kB [14,16,19,30]. It should be further noted that other miRNAs besides miRNA-34a (and miRNA-146a) may participate in regulating the expression of TREM2 at the post-transcriptional level.

### TREM2 is down-regulated in AMD retina

Human post-mortem retinal tissues were analyzed as ‘control whole retina’ and ‘macular region’ and ‘AMD whole retina and macular region’ and TREM2 protein abundance was assayed using Western blot analysis and/or ELISA (data not shown). At the level of protein TREM2 was found to be significantly down-regulated in AMD retina in both whole retina and the macular region to respectively 0.54- and 0.22-fold of controls (**Fig 1D–1F; data not shown**); hence both miRNA-34a increases and TREM2 deficits were found to be co-localized within the same retinal sample (**Fig 1A–1C).**

### Bioinformatics and complementarity analysis for the miRNA-34a-TREM2-mRNA-3’UTR interaction

Chromosomal assignments, gene structural schematics and DNA sequences obtained from publically accessible databanks, previously published reports or derived from DNA sequence analysis of human chromosomes 1p and 6p are further described in **Fig 2**. As indicated in **Fig 2** the controlled expression of TREM2 appears to be orchestrated by both transcriptional and post-transcriptional processes originating on 2 separate chromosomes: hsa-miRNA-34a encoded at chr1p36.15 and TREM2 is encoded at human chr6p21.1 [7,8,12,36–40].

### Relative abundance of TREM2 in control and stressed murine MG cells

Stressed MG cells have been used to study mechanistic aspects of inflammatory gene expression and innate-immune signaling in *in vitro* models of human CNS neurodegeneration, chelation and metal-induced neurotoxicity [12,19,21,41–43]. When compared to control MG cells at 3 days of culture, MG cells treated with TNFα alone showed a 2.1-fold decrease in TREM2 signal; MG cells treated with a negative control miRNA-34a (miRNA-NC) sequence show no significant deficits in TREM2 signal compared to control (**Fig 3A and 3B**). In contrast MG cells treated with a LNC-stabilized miRNA-34a sequence exhibited a significant 2.3-fold decrease in TREM2 signal (**Fig 3B**). Results from a typical Western blot analysis are shown for TREM2 and DAP12 abundance versus β-actin abundance in the same sample (upper panel), and in bar graph format (lower panel) **(Fig 3B).** Results are consistent with a TNFα- or miRNA-34a mediated down-regulation in the expression of TREM2 with no effects on the expression of DAP12. Importantly, as additional controls, a scrambled (**SC**) miRNA-34a sequence, other ‘pro-inflammatory miRNAs such as miRNA-125b, miRNA-146a or miRNA-183 showed no such significant interactive effects (**Figs 3B and 4); (**data not shown).

### Transfection of murine MG cells with a pLightSwitch TREM2-3’-UTR-luciferase reporter vector

Functional validation of a miRNA-34a-TREM2–3’UTR interaction in MG cells is shown in **Fig 4**; briefly, transfection of MG cells with a pLightSwitch TREM2-3’UTR-luciferase reporter (containing the entire 299 nt TREM2 mRNA 3’UTR; showed no significant effects on relative luciferase signal strength in controls, however, in the presence of a miRNA-34a mimic TREM2-3’UTR-luciferase signals were quenched ~4.5-fold of control and the results were highly significant (**Fig 4**). The scrambled control miRNA-34a-sc or miRNA-183 exhibited no effects in miRNA-34a-TREM2–3’UTR transfected MG cells. In agreement with previous reports, these data again suggest a productive miRNA-34a-mediated TREM2 mRNA interaction and a miRNA-34a-mediated down-regulation of TREM2 expression [10,12,21,44–53].

### miRNA-34a and TREM2 abundance in ROS-, IL-1β- or TNFα-stressed MG cells and treatment with PBN, CAY10512, CAPE or curcumin

ROS-, IL-1β- or TNFα stressed MG cells were found to induce miRNA-34a levels to 1.8-, 1.65- and 2.45-fold, respectively, over control levels while reducing TREM2 protein to 0.75-, 0.65- and 0.51-fold of control levels in the same sample (**Fig 5**). Because TNFα exhibited the strongest induction of miRNA-34a, the TNFα-mediated induction in murine MG cells was studied further. The use of the free radical scavenger PBN or the NF-kB inhibitors CAY10512, CAPE or curcumin were each found to lower ROS-, IL-1β- or TNFα-mediated inducibility of miRNA-34a while restoring TREM2 protein back to 0.85-, 0.72-, 0.88- and 0.76-fold of control levels, respectively (**Fig 5**). Interestingly, CAPE, an active component of propolis from honeybee hives and known to have anti-carcinogenic, anti-mitogenic, anti-inflammatory and immune-modulatory properties was found to be the most effective NF-kB inhibitor of TNFα-induced miRNA-34a used in these studies (**Fig 5**) [30,41,54–56].

### Phagocytosis of Aβ42 peptides; impairment in the presence of insufficient TREM2

We next studied the ability of MG cells to ingest or ‘phagocytose’ freshly prepared Aβ42 peptide monomers under various conditions in which miRNA-34a and TREM2 levels were altered (**Fig 6**). **Fig 6** (top row, panel **A-D**) shows staining for Aβ peptides, TREM2, DAPI and a merged view suggesting that that MG cells efficiently internalized Aβ42 peptides from the extracellular medium (yellow merge; λ_max_~580 nm; top row **panel D**). Briefly, stained sections displaying the intracellular Aβ42 in miRNA-34a treated or control MG cells were quantified for immunofluorescent intensity using SlideBook 5.0 and ImageJ software (NIH) as previously described [44]. The inclusion of a scrambled miRNA-34a sequence (miRNA-34a-sc; or other miRNAs, data not shown) had no significant effect on this process (middle row, **panel A-D**). However in the presence of a miRNA-34a mimic we observed: **(i)** significant decreases in the TREM2 signal (**Fig 6**; lowest row, panel **B**); **(ii)** a significantly reduced internalization of the Aβ42 peptides; and **(iii)** an increased amount of Aβ42 peptides that remained in the extracellular space (**Fig 6**; lower row, panel D).

## Discussion

Amyloidogenesis, the progressive deposition of pro-inflammatory Aβ peptides, is a prominent feature of several age-related neurological diseases of the human CNS including AMD. The dense, insoluble, pro-inflammatory lipoprotein-enriched lesions of AMD called drusen contain abundant βAPP-derived Aβ42 peptides at their core [1–3]. Via tandem beta- and gamma-secretase cleavage Aβ42 peptides are initially generated as monomers from βAPP, however due largely to their intensely lipophilic-hydrophobic character (21.4% valine-isoleucine) these monomers self-aggregate into higher order structures including dimers, oligomers and fibrils. These aggregates ultimately contribute to the formation of drusen that in part characterize the dry form of AMD [2–5,31–36]. The CNS and retina have evolved highly effective MG cell-mediated clearance mechanisms to phagocytose Aβ42 peptide monomers from the extracellular space using transmembrane-associated glycoprotein sensors such as TREM2. *When Aβ42 peptide monomers are overproduced*, *or if deficits in TREM2 and other components of the phagocytic system fail to clear Aβ42*, *amyloids accumulate and self-aggregate with pro-inflammatory and pathological consequences* [6–10,37–41]. Interestingly, the transition of Aβ42 peptide monomers into higher-order structures may be especially stimulated in the presence of physiologically realistic (low nanomolar) concentrations of neurotoxic metals including metal sulfates, such as aluminum sulfate [42,43]. Deficits in other immunoglobulin-like MG transmembrane glycoproteins, including the 67 kD CD33/Siglec-3 (sialic acid-binding immunoglobulin-like lectin-3; gp67; chr 19q13.3) sialoadhesion protein have also been recently described as contributing to the impairment in Aβ42 peptide clearance from the brain, and decreased expression of CD33/Siglec-3 has recently been reported in the peripheral mononuclear cells of AD patients [45].

**Fig 7:**
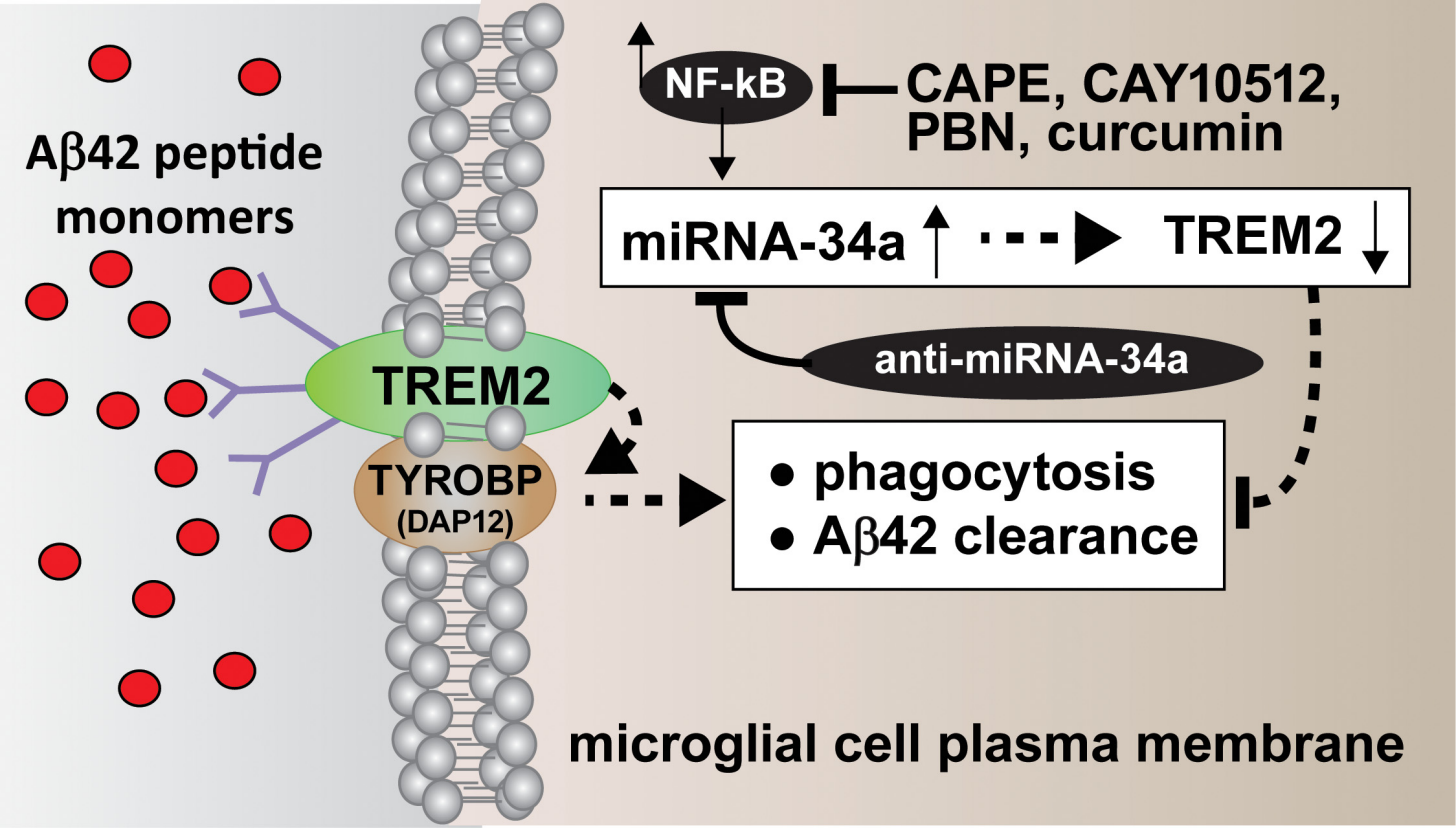
**Schematic of the structure and function of an NF-kB-regulated, miRNA-34a-mediated TREM2 sensor-receptor circuit down-regulated in AMD and in stressed MG cells;** TREM2 is a variably glycosylated, single pass, integrated transmembrane sensor-receptor (green oval; deglycosylated MW ~26 kDa) embedded in the MG plasma membrane [37–39,46–53,85]; TREM2-mediated signaling via the tripartite TYROBP (DAP12) accessory receptor (brown oval; MW ~12 kDa) results in Aβ42 peptide engulfment, phagocytosis and ultimately, clearance of Aβ42 peptides (red spheres) from the extracellular space; ***TREM2 appears to be able to deal effectively with Aβ42 peptide monomers*, *however encounter difficulty ingesting Aβ42 peptide dimers*, *oligomers or higher order structures*** (**Fig 6) (**and unpublished observations); ***insufficient TREM2 may be in part responsible for the inability to adequately phagocytose Aβ42 peptide monomers resulting in their self-aggregation in the extracellular space***; neurotoxic metals (such as aluminum) may contribute to the aggregation of external Aβ42 peptide monomers into higher order structures while also up-regulating additional miRNA-34a via NF-kB activation [21,42,43]; importantly, TYROBP (DAP12) protein levels were found to be unchanged in sporadic AMD or in stressed MG cells (unpublished observations); see (**Fig 3**); TREM2 mutations may affect MG cell’s ability to phagocytose [6–14;85]. **Inset:** the NF-kB-induced, pro-inflammatory miRNA-34a is found to be significantly increased in AMD retina and in stressed MG cells; miRNA-34a targeting of the TREM2 mRNA 3’UTR appears to be in part responsible for the down-regulation of TREM2 expression (see **Figs 2 and 3**); because miRNA-34a is an NF-kB-regulated transcript inducible by ROS and pro-inflammatory cytokine stressors from outside of the cell, free radical scavenging (PBN), anti-NF-kB (CAPE, CAY10512, curcumin) and/or anti-miRNA-34a (AM-34a) strategies (elongated black ovals) or combinatorial strategies may be clinically useful in the restoration of TREM2 and ‘homeostatic’ phagocytosis.

TREM2 is a ~26 kD, variably glycosylated single pass transmembrane-spanning stimulatory sensor-receptor of the immune-globulin/lectin-like TREM gene superfamily highly enriched in abundance in MG cell plasma membranes [6–15] (**Fig 7**). TREM2 appears to be involved in multiple aspects of bacterial and neurotoxin sensing, innate- and adaptive-immunity, axon guidance, semaphorin signaling and lipid sensing [6–14,46–51]. The TREM superfamily also includes a sepsis-associated TREM1 and a soluble form of TREM2 which may extend TREM2 activities well beyond the cells in which they were initially generated [37–39]. Interestingly TREM-2 appears to recognize and bind repeated anionic motifs on yeast, and gram-positive and/or gram-negative bacteria, but how Aβ42 peptides are recognized by TREM2 extracellular domains is not well understood [36–39].

Some preliminary work on TREM2 neurobiology, TREM2-mediated Aβ42 peptide ingestion, TREM2 induction and altered TREM2 signaling in the progressive neocortical degenerative disease AD have recently been reported [6–15,21,42,46–56]. Loss-of-function mutations for TREM2 such as the common rs75932628 variant (encoding R47H) have been associated with deficiencies in the innate-immune system, axonal and synaptic abnormalities, deficits in phagocytosis and progressive dementia in neurodegenerative diseases including *PLOSL (*Nasu-Hakola disease) as well as more recently in the sporadic forms of familial ALS and AD [6–14,49–53,85]. Coding variants in TREM2 have been shown to increase the risk for AD in the elderly of European-American and African American descent but this risk is significantly diminished in AD patients from Chinese and Japanese populations [49–55]. Importantly, all retinal tissues examined in this study were derived from sporadic dry AMD cases with no known familial association.

microRNAs are a fascinating class of small, non-coding 21–25 nt ssRNAs and are the smallest carriers of highly specific genetic information so far discovered, remarkably similar in structure and pathogenic mechanism to ssRNA viroids that cause unusual progressive degenerative diseases in plants [54–58]). The major mechanism of miRNA actions are to recognize complementary RNA sequences in the 3’UTR of their target mRNAs and down-regulate or quench expression of that target mRNA (**Figs 2 and 4**) [55–58]. Several inducible miRNAs such as miRNA-34a are under transcriptional control by the pro-inflammatory transcription factor NF-kB in the CNS and other tissues, so up-regulated NF-kB, via increasing specific miRNA abundances, may ultimately act as an important down-regulator of the expression of multiple sporadic AMD-relevant genes [22,25,29,56,58,59]. In this study we initially characterized total miRNA expression in whole AMD retina, in a macular-enriched region of the AMD retina, and in ROS- (peroxide), IL-1β- and/or Aβ42-treated MG cells and found a significant up-regulation of an NF-kB-sensitive miRNA-34a closely linked to a down-regulation in a miRNA-34a mRNA target encoding TREM2 within the same tissue and cell samples. Specific up-regulation of miRNA-34a-signalling has been previously associated with at least **15** neurological, neuro-immune, neuro-inflammatory and/or neurodegenerative pathologies including **(i)** diseased spinal cord tissues in amyotrophic lateral sclerosis [11]**; (ii)** peripheral blood mononuclear cells and blood plasma in sporadic AD patients [60,61]; **(iii)** altered immunological signaling associated with multiple sclerosis [62]; **(iv)** autoimmune encephalomyelitis [37,38]; **(v)** progressive neurotrophic deficits, including dysfunctional Bcl-2 signaling, in transgenic murine models of AD (TgAD) including the APPswe/PSDeltaE9 model [63]; **(vi)** altered synaptotagmin-1 and syntaxin-1A signaling, synaptogenesis and neurite outgrowth [64]; **(vii)** repression in the expression of several genes involved in cell survival and oxidative defense pathways such as Bcl2 and SIRT1 [61]; **(viii)** accelerated aging of the murine brain [65]; **(ix)** deficient immune- and phagocytotic-responses in progressive inflammatory degeneration in cardiovascular disease [66]; **(x)** aging of the vasculature and cellular senescence [66–68]; **(xi)**; the mis-regulation of p53-regulated genes contributing to DNA damage, p53-mediated apoptosis and mitotic catastrophe [69]; **(xii)** astroglial cell proliferation, gliosis and tumor progression [70]; **(xiii)** lower mini-mental state examination (MMSE) scores linked to elevated miRNA-34a in the blood plasma of AD patients [61]; **(xiv)** major depressive disorder (MDD) [67]; and **(xv)** progressive inflammatory neurodegeneration and epileptiform activities associated with epilepsy and the early stages of AD [12,71]. The involvement of miRNA-34a in epilepsy and AD is particularly interesting because of the overlapping neuropathology of these two disorders with respect to seizure frequency and cognitive decline first apparent in the earliest stages of each disease [70–72].

The current work provides at least 8 lines of evidence to suggest that an NF-κB-sensitive miRNA-34a acts as a repressor of its TREM2 mRNA target: **(i)** up-regulation of miRNA-34a in AMD retina corresponds to down-regulation of TREM2 in the same retinal tissues and stressed MG cells (**Figs 1, 2, 3 and 4); (ii)** human miRNA-34a is highly complementary to the human TREM2 mRNA 3’UTR (~88% homology between the miRNA-34a 5’ region and the TREM2-3’UTR seed sequence; structural stability ~-22 kcal/mol; **Figs 2 and 4**); **(iii)** when a stabilized miRNA-34a, but not negative control (**NC**) or scrambled (**SC**) miRNAs are added to MG cells in culture there is a significant down-regulation of TREM2 signal **(Fig 3)**; **(iv)** ROS, IL-1β- and TNFα-stressed MG cells exhibit up-regulation of miRNA-34a corresponding to down-regulation of TREM2 in the same sample (**Fig 5**); **(v)** the free radical scavenger PBN reduces TNFα-mediated up-regulation of miRNA-34a and significantly restores TREM2 levels (**Figs 3 and 5**); **(vi)** the substituted *trans*-stilbene resveratrol analog and NF-κB inhibitors CAPE, CAY10512 and curcumin also strongly inhibited miRNA-34a promoter-luciferase reporter activity (**Figs 4 and 5**); **(vii)** an anti-miRNA-34a (AM34a) specifically directed against miRNA-34a was found to restore TREM2 protein to near control levels in stressed MG cells (**Fig 5**); and **(viii)** an exogenously added miRNA-34a mimic (but not **NC** or **SC** miRNAs) were found to down-regulate TREM2 and impair MG cells from phagocytosing Aβ42 peptide monomers (**Fig 6**). Taken together the results suggest involvement of an oxidative stress- and NF-kB-inducible miRNA-34a in the regulation of TREM2 and TREM2-mediated phagocytic activities in the CNS. Indeed the contribution of NF-kB and the potential use of NF-kB inhibitors in managing the up-regulation of NF-kB-sensitive pro-inflammatory miRNAs with pathological consequences has been extensively addessed in several recent reports [10,12,20,41,56–59]. Although we examined miRNA-34a and TREM2 levels in 21 carefully selected human sporadic AMD and age-matched control retina other miRNAs or other genetic factors may play ancillary roles in phagocytosis in this retinal disease, especially in diverse human population sets [73–75]. MG cell age may be a variable factor with older MG cells exhibiting reduced immune responses and phagocytic capabilities (unpublished observations) [19–22]. At this point while the NF-kB-miRNA-34a-TREM2 circuit appears to be dysfunctional in AMD retina and in ROS- and cytokine-stressed MG cells we cannot exclude that other brain-enriched miRNAs have ancillary control on TREM2 mRNA stability and expression in the retina and CNS.

In summary, dysfunctional phagocytosis in AMD retina and Aβ42 peptide aggregation into drusen have been attributed to disease-specific deficits in Aβ42 clearance. These current data are the first to suggest that the pathological up-regulation of miRNA-34a abundance in AMD retina and in stressed MG cells are linked to a functional repression of TREM2 expression and bioavailability. The results further suggest that the mis-regulation of specific miRNAs in AMD contributes to amyloidogenesis, a known driver of inflammatory neuropathology and disruption of normal innate-immune and inflammatory responses. It appears that Aβ42 peptide generation and clearance are a carefully orchestrated, and continually maintained homeostatic mechanism, in the CNS that when upset leads to amyloidogenic and pathological consequences. While healthy MG appear to deal relatively easily with Aβ42 peptide monomers they appear to encounter difficulty: **(i)** when Aβ42 peptide monomers are generated in excess, or **(ii)** when amyloid dimers and higher order oligomeric Aβ42 peptide structures preclude TREM2-mediated phagocytosis. This further suggests that Aβ42 monomer phagocytosis is in a steady state and that any factors that promote aggregation of Aβ42 monomers, including excessive Aβ42 peptide generation, or coalescence via neurotoxic metals or other factors, may easily upset this sensitive homeostatic balance. **Fig 7** is a highly schematicized depiction of the actions of an NF-kB-regulated, miRNA-34a-mediated TREM2 sensor-phagocytosis system down-regulated in stressed MG cells and AMD. Signaling via the TREM2/tyrosine binding protein/DNAX-activating protein of 12 kDa (TYROBP/DAP12) receptor complex results in phagocytosis and ultimately, clearance of Aβ42 peptides from the extracellular medium [13,76–81]. Interestingly, TREM2 knockdown or knockout mice exhibit attenuated immunological responses and/or increases in age-related neuroinflammatory biomarkers [79,80]. It has also recently been shown that TREM2 deficiencies exacerbate tau pathology and promote neurodegenerative change and spatial learning deficits in P301S tau transgenic mice [81]. Importantly no deficits in the TYROBP/DAP12 adaptor protein have been observed in stressed MG cells or in AMD retina (**Fig 3**; unpublished observations).

Lastly, using multiple interdependent techniques it should be mentioned that miRNA-34a has also been recently found to be significantly increased in limbic regions of the AD-affected brain, in AD monocytes and in several amyloid overexpressing transgenic murine models of AD (unpublished observations) [10,12,48,60,61,63,65]. A dysfunctional TREM2 sensor-receptor, through a loss-of-function mutation in familial AD, may have the same net end result as an insufficient amount of a functional TREM2 phagocytosis-sensor with both pathological scenarios resulting in a significant impairment in the ability to effectively phagocytose and clear Aβ42 peptides. The data reported here are the first to indicate that the orchestrated interaction of at least two independent gene products on two different human chromosomes—miRNA-34a at chr1p36.22 and TREM2 at chr6p21.1—are required to modulate TREM2 activities, the sensing of potentially hazardous amyloidogenic molecules in the extracellular space, and the phagocytosis and clearance of retinotoxic debris to maintain functional homeostasis in the retina. Notably, this type of multigenic miRNA-34a-mediated regulation would escape detection by standard GWAS-based genomic analysis. Because miRNA-34a is encoded as an NF-kB-sensitive transcript, anti-NF-kB and/or anti-miRNA strategies and/or combinatorial approaches, perhaps with other targeted anti-inflammatory therapies, may be useful in the clinical management of AMD and in other disorders of the CNS with an amyloidogenic component [79–85].
