# Correction: microRNA-34a-Mediated Down-Regulation of the Microglial-Enriched Triggering Receptor and Phagocytosis-Sensor TREM2 in Age-Related Macular Degeneration

**DOI:** 10.1371/journal.pone.0153292

**Published:** 2016-04-05

**Authors:** The PLOS ONE Staff

There are errors in the Abstract. All references to NF-B should instead be to NF-kB. The publisher apologizes for this error.
